# Mahanimbine Improved Aging-Related Memory Deficits in Mice through Enhanced Cholinergic Transmission and Suppressed Oxidative Stress, Amyloid Levels, and Neuroinflammation

**DOI:** 10.3390/brainsci12010012

**Published:** 2021-12-23

**Authors:** Vasudevan Mani, Nur Syamimi Mohd Azahan, Kalavathy Ramasamy, Siong Meng Lim, Abu Bakar Abdul Majeed

**Affiliations:** 1 Department of Pharmacology and Toxicology, College of Pharmacy, Qassim University, Buraydah 51452, Saudi Arabia; 2 Collaborative Drug Discovery Research (CDDR) Group and Brain Degeneration and Therapeutics Group, Faculty of Pharmacy, Universiti Teknologi MARA (UiTM), Cawangan Selangor, Kampus Puncak Alam, Bandar Puncak Alam 42300, Malaysia; mimi_azahan@yahoo.com (N.S.M.A.); kalav922@uitm.edu.my (K.R.); lim219@uitm.edu.my (S.M.L.); abubakar@uitm.edu.my (A.B.A.M.)

**Keywords:** mahanimbine, aging, memory, acetylcholine, neuroinflammation, β-amyloid, antioxidants

## Abstract

*Murraya koenigii* leaves contain mahanimbine, a carbazole alkaloid, reported with improving cholinergic neuronal transmission and reducing neuroinflammation in the CNS. The current research investigated the effects of mahanimbine on age-related memory deficits, oxidative stress, cholinergic dysfunction, amyloid formation, and neuroinflammation in aged mice (16 months old). Mahanimbine was administered (1 and 2 mg/kg, p.o.) daily to groups of aged mice for 30 days. The Morris water maze (MWM) task was performed to study spatial learning (escape latency (EL) and swimming distance (SD)) and memory (probe test). The levels of malondialdehyde (MDA), glutathione (GSH), acetylcholine (ACh), acetylcholinesterase (AChE), β-amyloid (Aβ_1-40_ and Aβ_1-42_), β-secretase (BACE-1), as well as neuroinflammation markers (total cyclooxygenase (COX) and COX-2 expression), were measured from the isolated brain. Mahanimbine reduced the EL time and SD in the MWM test. From the probe trial, the mahanimbine-treated group spent more time in the targeted quadrant related to the age-matched control, which indicated the enhancement of memory retention. From the biochemical tests, the treatment decreased MDA, AChE, Aβ_1-40_, and Aβ_1-42_, BACE-1, total COX activity, and COX-2 expression. It also raised the brain GSH and ACh levels in aged mice compared to age-matched control. These results have supported the reversal of memory dysfunctions by mahanimbine in aged mice and hypothesized that it could be a potential target to treat age-related neurodegenerative disease.

## 1. Introduction

The global population of aging people over 60 years old has grown from 8.5% in 1980 to 12.3% in 2015, projected to 21.5% in 2050, according to the report of United Nations, 2015 [1]. Aging is a normal component of life that refers to an organism’s physiological function gradually deteriorating through time [1]. Memory impairment related to aging is a common feature of the aging brain, and it is linked to declines in learning and memory, synaptic plasticity, and adult neurogenesis [2]. Oxidative stress by reactive oxygen species (ROS) has been reported as a vulnerable cause in the aging process. An early study has found that cognitive decline is related to the excessive development of ROS in aging mice brain tissues [3]. Meanwhile, malondialdehyde (MDA), an end product from the peroxidation of polyunsaturated fatty acid, is targeted as a typical marker for oxidative stress. Because the brain is extremely susceptible to this condition, it has contributed to cognitive impairment. Therefore, antioxidant systems play a role in the defense system against oxidative stress conditions [4,5]. Besides, defects in the cholinergic system can affect synaptic transmission and are characterized by the loss of synapses in older mice. Acetylcholine (ACh) is an important neurotransmitter that mediates transmission at cholinergic neurons and coordinates various cognitive functions. As a result, monitoring changes in extracellular neurotransmitter levels in specific brain locations are thought to be a useful method for identifying the neuronal systems engaged in specific memory processes [6].

Besides, in Alzheimer’s disease (AD), the process of aging is linked to the development of amyloid plaques, which is one of the illness’s pathogenic features. The accumulation of brain Aβ peptide levels resulted in cognitive dysfunction, including memory impairment [7]. Aβ peptide was formed through the amyloidogenic pathway of proteolytic cleavage at the site of amyloid precursor protein (APP) arbitrated through β-secretase (BACE-1) followed by γ-secretase enzymes [8]. In addition, inflammation has become another factor closely linked to major degenerative diseases in the elderly [9]. Neuroinflammatory reactions appear to be most evident in the brain region that mediates contextual and spatial memory, which could lead to memory loss in the elderly [10]. The conversion of arachidonate to prostaglandin catalyzes by cyclooxygenase (COX) is a crucial step to initiate the inflammatory process. The COX-1 and COX-2 are two distinct isoforms from COX. Principally, isoform COX-2 is induced at the site of inflammation by pro-inflammatory cytokines [11].

*Murraya koenigii* Linn (*M. koenigii*) (Family Rutaceae) is often known as curry leaves. The whole plant of *M. koenigii* consists of many carbazole alkaloids, including mahanimbine, koenimbine, mahanine, bismurrayafoline, murrayanol, isomahanine, bismahanine, euchrestine, bispyrafoline, and girinimbine [12]. Amongst these compounds, mahanimbine is majorly isolated from *M. koenigii* leaves [13]. It has been reported to reduce total cholesterol as well as elevate HDL levels through its hypoglycemic effects in diabetic-induced rats [14]. A group of carbazole alkaloids has also been reported to exhibit antioxidant, anti-inflammatory, anti-tumor, and anti-viral activities [15,16]. To support this, our results have verified that the leaves of *M. koenigii* have an anti-amnesic potential when administered to groups of mice continuously for thirty days [17]. Moreover, *M. koenigii* leaves’ total alkaloidal fraction reduced oxidative stress and improved the cholinergic activity in the aged mouse brain [18]. Recently, our data showed that oral administration of mahanimbine reversed the memory impairment that was induced by lipopolysaccharides (LPS) in mice. The same treatment resulted in improvement of cholinergic transmission and attenuation of neuroinflammation as well as amyloid levels in the LPS-induced mouse brain [19]. However, the effect of mahanimbine related to aging-induced memory is not well documented. Therefore, the current objectives focused on evaluating its potential in enhancing spatial learning and memory via suppressing oxidative stress and declining deposition of β-amyloid and neuroinflammation in aged mice.

## 2. Materials and Methods

### 2.1. Extraction and Isolation of Mahanimbine

Fresh leaves of *M. koenigii* were purchased at a local market in Puncak Alam, Malaysia. The collected leaves were authenticated by a taxonomist from Forest Research Institute, Malaysia, and a voucher specimen (PID 24101011) was submitted to the herbarium. The isolation method of mahanimbine from *M. koenigii* leaves followed our previous reports [19,20]. The isolated mahanimbine was then confirmed with spectral data from NMR, HPLC, and mass spectra.

### 2.2. Experimental Animals

A group (*n* = 6) of young (4 months) and three groups (*n* = 6) of aged (16 months) C57BL/6N male mice collected from Laboratory Animal Facility and Management (LAFAM), Universiti Teknologi MARA (UiTM), Malaysia, were used in this study. The mice were kept in polyacrylic cages and retained at ambient temperature (21–25 °C). All animals were given free access to the normal diet (gold coin pellet) and water. After the collection, five days’ acclimatization procedure was followed before the experiment. The time of the behavioral experiments was fixed between 08:00 and 18:00 h. Additionally, the procedure of the present experiment was accepted by the Research Committee on Ethical Use in Research (approval number: UiTM Care-372014), UiTM, Malaysia.

### 2.3. Mahanimbine Administration

**Figure 1:**
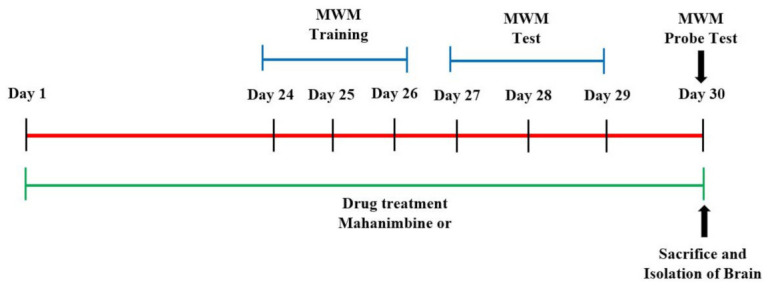
Timeline administration of drug, behavioral assessments, and isolation of brain samples. The groups of young and aged mice were administered mahanimbine (1 or 2 mg/kg, p.o.) or vehicle for 30 days. For MWM analysis, the training sessions were conducted from days 24 to 26. The memory analysis was assessed from day 29 to 30. On day 30, after the probe test, all the animals were sacrificed, and brain tissues were isolated for further biochemical estimation.

A total of 18 aged mice were divided into three groups (*n* = 6). Among them, two selected groups of the aged mice were administrated daily oral gavage of mahanimbine at doses of 1 and 2 mg/kg, respectively, for 30 days (day 1–day 30). The 0.5% *w*/*v* of carboxymethylcellulose (CMC) was used as a vehicle for preparing the mahanimbine doses (1 or 2 mg/kg). The remaining group of aged mice and young mice (*n* = 6) served as young and aged controls, respectively, and were administered with vehicle (0.5% *w*/*v* of CMC) as above. The behavioral test was employed between day 24 to day 30 (Figure 1). All the animals were sacrificed on day 30 after the end of the probe test, and the brain sample was isolated for further biochemical estimations.

### 2.4. Behavioral Assessment of Memory by the Morris Water Maze (MWM)

The MWM protocol was carried out to follow the procedure outlined by Mohd Azahan et al. (2020) [19]. A black round pool (100 cm diameter × 35 cm height) with a hidden platform (4.5 cm diameter × 14.5 cm height) was utilized. The entire pool was separated into four quadrants (NE, SE, NW, and SW), and the platform was located at the midpoint of a target quadrant (SW). The MWM assessment (days 24–30) was conducted continually for seven consecutive days (3 days of training, 3 days of actual task, 1 day of probe test) during the treatment. For the training session (days 24–26), animals were trained three trials twice per day with a visible platform from different quadrants to find the hidden platform at a target quadrant (SW). In the case of any failure in locating the platform in 60 s, the animal was manually guided to find the platform. The actual experiments were conducted over the next three days (days 27–29), with each animal swimming from a set beginning point (NE). The escape latency (EL), swimming speed, and escape distance were documented using a tracking system (SMART-LD Program, Stafford St La Puente, CA, USA). A probe test session was conducted on day 30, and each mouse was permitted to swim freely without any platform for 60 s. A tracking system was used to record the proportion of time spent by each animal in the targeted quadrant (SW) as memory retention.

### 2.5. Collection of Brain Samples

On day 30, the brain tissues were isolated from mice. For biochemical estimation, half of the brain was stored in cold PBS; the other half was kept in RNA for later gene expression analysis. A glass WiseStir Homogenizer (INCO instruments, Ambala, India) was used to homogenize the brain kept in PBS. The supernatant was collected after the homogenized brain was centrifuged for 10 min at 4000 rpm and 4 °C. For biochemical examination, the supernatant was maintained at −80 °C.

### 2.6. Evaluation of Malondialdehyde (MDA) and Glutathione (GSH) Levels

Using commercially available kits, the MDA and GSH levels were analyzed in brain samples (Cayman Chemical Company, Ann Arbor, MI, USA). MDA and GSH levels were measured in nanomoles/milligram of protein and micromoles/milligram of protein, correspondingly. Bradford technique was followed to estimate the protein content in the brain tissues.

### 2.7. Estimation of Acetylcholine (ACh) and Acetylcholinesterase (AChE) Activities

The ACh and AChE levels in the brain homogenate were analyzed using commercialized kits (BioAssay System, Hayward, CA, USA), which were EnzyChrom^TM^ Acetylcholine Assay kit and QuantiChrom^TM^ Acetylcholinesterase Assay kit, respectively. The intensity of color formation was detected in a microplate reader at 570 and 412 nm, respectively. The level of ACh obtained was expressed as µM, whereas AChE activity was expressed as U/L.

### 2.8. Measurement of β-Amyloid Levels

The ELISA kits from Cloud Clone Corp (Katy, TX, USA) were used to evaluate the levels of Aβ_1-40_ and Aβ_1-42_ in brain homogenates. A microplate reader set to 450 nm was used to measure the color formation. The concentration of Aβ_1-40_ and Aβ_1-42_ in the sample was inversely related to the color intensity. The results were represented in terms of pg/mL of total protein.

### 2.9. Assessment of β-Secretase (BACE-1) Activity in Mouse Brain

BACE-1 activity in the homogenate was quantified using a commercialized kit (SensoLyte^®^ β-Secretase Assay Kit from AnaSpec, Fremont, CA, USA). The detection of the kit is based on a peptide, which is cleaved by BACE-1, and the signal is detected using a spectrophotometer with excitation/emission at 490/520 nm.

### 2.10. Measurement of Total Cyclooxygenase (COX) Activity

The activity of total COX in the brain homogenate was estimated using an ELISA kit (Cayman Chemical Company, Ann Arbor, MI, USA). The color formation was directly proportional to the total COX activity. A microplate reader was utilized to read the absorbance at 590 nm.

### 2.11. Gene Expression of BACE-1 and COX-2

The expression of BACE-1 and COX-2 in isolated brain RNA was analyzed using RT-PCR. The three major steps involved were: RNA extraction, conversion of RNA to cDNA, and real-time polymerase chain reaction (RT-PCR). All the steps involved kits provided by Qiagen Valencia, CA, USA. Firstly, RNA was extracted from the brain sample preserved in the RNA. In this step, approximately 100 mg of brain tissue sample was homogenized using a TissueRuptor in 1 mL of QIAzol lysis reagent and incubated for 5 min at room temperature. The mixture of brain lysate and QIAzol lysis reagent was added with chloroform and shaken vigorously for 15 s, followed by incubation at room temperature for 2–3 min. The mixture was centrifuged at 12,000× *g* for 15 min at 4 °C, and the three phases formed a colorless phase (upper phase), which consisted of RNA and was collected with 1 volume of 70% ethanol added to it. Next, 700 μL of the sample was transferred into a spin column and centrifuged at room temperature at 8000× *g* for 15 s; the flow-through was discarded. Following the addition of 700 μL of buffer RW1 (provided in the kit) and centrifugation for 15 s at 8000× *g*, the flow-through was discarded. The step was continued with the addition of 500 μL buffer RPE (provided in the kit) and centrifugation for 15 s at 8000× *g*. This step was repeated twice to wash any organic contamination in the RNA. Lastly, RNA was eluted by adding 50 μL of RNAse-free water and centrifuged for 1 min at 8000× *g*. The concentration of RNA was measured using a nanodrop (nanodrop 2000c, Thermofisher Scientific, Waltham, MA, USA). The extracted RNA was then converted into cDNA, which was utilized to perform RT-PCR. Firstly, 14 μL of template RNA was added with the 6 μL reverse-transcription master mix (1 μL of Quantiscript reverse transcriptase, 4 μL of Quantiscript RT buffer, 1 μL of RT primer mix). After mixing, it was incubated for 15 min at 42 °C, followed by incubation at 95 °C for 3 min to inactivate the Quantiscript reverse transcriptase. The product formed was cDNA, and finally, RT-PCR was conducted. The RT-PCR conditions were fixed as 95 °C for 5 min followed by 40 cycles at 95 °C for 10 min and 60 °C for 30 min. The expression of BACE-1 and COX-2 genes were quantified and normalized against the two house-keeping genes (β-actin and GAPDH). The primers of nucleotide sequences were based on mouse BACE-1 (front 5′-GCATGATCATTGGTGGTATC-3′: reverse 5′-CCATCTTGAGATCTTGAC-CA-3′) and COX-2 (forward 5′-GTGTGCGACATACTCAAGCAGGA) cDNA sequences. -3′: reverse 5′-TGAAGTGGTAACCGCTCAGGTG-3′), GAPDH (forward 5′-TGACAGGATGCAGAAGGAGA-3′: reverse 5′-GCTGGAAGGTGGACAGTGAG-3′), and -actin (forward 5′-TGACAGGATGCAGAAGGAGA-3′: reverse 5′-GCTGGAAGGTGGACAGTGAG-3′). Fluorescence measurements were obtained, and Rotor-Gene 6000 software was used to evaluate them (Qiagen, Hilden, Germany). Results were presented as the levels of expression following normalization to the housekeeping gene, which were B-actin and GAPDH using the comparative CT (threshold cycle) approach. Equation (1) was used to calculate the gene expression of the sample. The CT value of each sample was obtained based on the standard curve that was generated for each gene (target gene and reference gene).

ΔΔCT = ΔCT sample − ΔCT control(1)

where

ΔCT sample: CT value of target gene − CT value of reference gene

ΔCT control: CT value of target gene − CT value of reference gene

### 2.12. Statistical Analysis

Experimental data were presented as mean ± SEM. One-way ANOVA procedure (Graph Pad version 9, GraphPad Software Inc., La Jolla, CA, USA) and Tukey–Kramer post hoc test were utilized to categorize statistical variations. Significant was defined as a probability value of 0.05.

## 3. Results

### 3.1. Mahanimbine-Enhanced Memory in Aged Mice

The spatial learning and memory ability of mahanimbine in the mouse model was examined using the MWM test. Escape latency (EL), escape distance (ED), and time spent in the target quadrant were measured. Moreover, locomotor activity was also considered based on the average swimming speed of mice.

**Figure 2:**
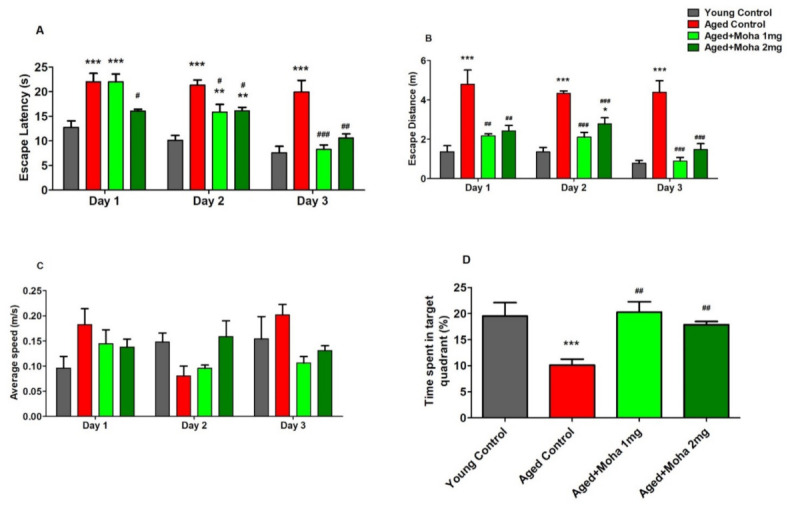
Effect of mahanimbine on (**A**) escape latency, time taken to find the hidden platform. (**B**) Escape distance, distance traveled from starting point until finding the hidden platform; (**C**) average speed, swimming speed of animal to find the hidden platform; (**D**) probe test, aged mice’s percentage time spent in the target quadrant using Morris water maze test. There were significant improvements in escape latency and escape distance with mahanimbine in aged mice. Additionally, treatment increased the time spent in the target quadrant of aged mice. All data were expressed as mean ± SEM (*n* = 6). One-way ANOVA (day 1: *F*(3,20) = 11.74, *p* < 0.001, day 2: *F*(3,20) = 17.38, *p* < 0.001 and day 3: *F*(3,20) = 14.88, *p* < 0.001 for escape latency; day 1: *F*(3,20) = 12.22, *p* < 0.001, day 2: *F*(3,20) = 28.62, *p* < 0.001, and day 3: *F*(3,20) = 23.15, *p* < 0.001 for escape distance; day 1: *F*(3,20) = 1.939, *p* > 0.05, day 2: *F*(3,20) = 3.467, *p* > 0.05, and day 3: *F*(3,20) = 2.497, *p* > 0.05 for average speed; *F*(3,20) = 7.185, *p* < 0.01 for probe test followed by Tukey–Kramer multiple comparisons test. ** *p* < 0.01 and *** *p* < 0.001 in comparison with young control, ^#^ *p* < 0.05, ^##^ *p* < 0.01, and ^###^ *p* < 0.001 in comparison with aged control.

Figure 2A shows that the aged control group had markedly longer EL from day 1 to day 3 (22.05 ± 1.70 s, 21.35 ± 1.85 s, 19.96 ± 2.34 s; *p* < 0.001; respectively) when compared to the young control (12.75 ± 1.30, 10.12 ± 0.98, 7.60 ± 1.31; respectively). It revealed that aged mice established a substantial deficit in learning and memory function in the MWM test. However, oral treatment with different dosages (1 and 2 mg/kg) of mahanimbine showed a reversal of the aforementioned changes in EL of aged mice. The EL values for the 1 mg/kg mahanimbine group were 22.01 ± 1.60 s, 15.88 ± 1.54 s (*p* < 0.01), and 8.26 ± 0.93 s (*p* < 0.001) for days 1, 2, and 3, correspondingly compared with the aged control, whereas the EL values for the 2 mg/kg mahanimbine group were 16.09 ± 0.33 s (*p* < 0.05), 17.14 ± 0.57 s (*p* < 0.05), 10.57 ± 0.86 s (*p* < 0.01), respectively, when paralleled to the aged control. On day 3, the EL of both groups (1 and 2 mg/kg) of mahanimbine was not significantly different from the young control.

Figure 2B represents the effect of mahanimbine on the distance traveled from the starting point until finding the hidden platform. The aged control group traveled the longest distance before finding the hidden platform on days 1, 2, and 3 (4.79 ± 0.73 m, 4.34 ± 0.11 m and 4.38 ± 0.59 m; *p* < 0.001; respectively) when related to the young control (1.36 ± 0.31 m, 1.35 ± 0.24 m, and 0.78 ± 0.14 m; respectively). Aged mice that were fed 1 mg/kg of mahanimbine significantly reduced the ED (2.17 ± 0.11 m (*p* < 0.01), 2.10 ± 0.24 m (*p* < 0.001), 0.89 ± 0.18 (*p* < 0.001); respectively) for day 1 until day 3 as matched to the aged control. A significant parallel decline in ED was observed in mice administered 2 mg/kg of mahanimbine for days 1, 2, and 3 (2.43 ± 0.28 (*p* < 0.01), 2.78 ± 0.32 (*p* < 0.001), 1.47 ± 0.30 (*p* < 0.001); respectively).

With respect to the average swimming speeds during the task, no differences were observed among the groups (Figure 2C). This indicates that mahanimbine did not alter any locomotor activity related to motor function in aged mice.

After 24 h of the actual task, a probe test session (day 30) was conducted to evaluate the memory retention of animals. The aged control group (Figure 2D) spent less time in the target quadrant (10.10 ± 1.14% (*p* < 0.001)) and showed a significantly reduced time spent in the targeted quadrant compared to the young control (19.53 ± 2.56%), whereas aged mice treated with 1 mg/kg (20.27 ± 4.84%, *p* < 0.01) and 2 mg/kg (17.85 ± 1.56%, *p* < 0.05) of mahanimbine by oral gavage showed a significantly longer time spent in the targeted quadrant compared with the aged control.

### 3.2. Effect of Mahanimbine on MDA and GSH Levels in the Aged Mouse Brain

**Figure 3:**
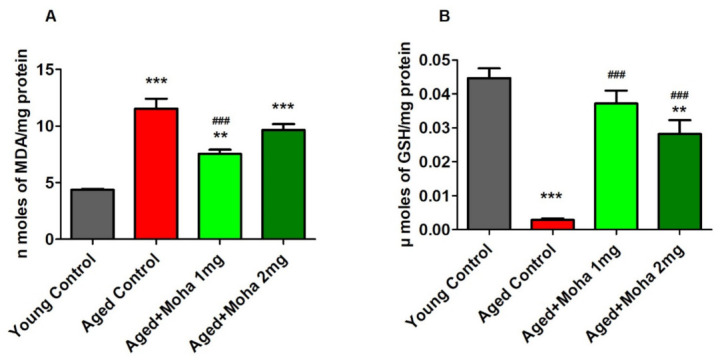
Effects of mahanimbine on (**A**) MDA and (**B**) GSH levels in brain homogenates of the aged mice mouse model. Administration of mahanimbine significantly reduced MDA levels and increased GSH levels in aged mice brains. All data were expressed as mean ± SEM (*n* = 6). One-way ANOVA (*F*(3,20) = 32.48, *p* < 0.001 for MDA level and *F*(3,20) = 33.39, *p* < 0.001 for GSH level) followed by Tukey–Kramer multiple comparisons test. ** *p* < 0.01 and *** *p* < 0.001 in comparison with young control; ^###^ *p* < 0.001 in comparison with aged control.

The MDA levels in the aged control (Figure 3A) were considerably higher (11.54 ± 0.88 µM; *p* < 0.001) related to the young control (4.37 ± 0.09 µM). The increase in the MDA level in the brain homogenate of aged mice reflected a higher LPO activity, which indicated an elevation in oxidative stress in the brain tissue. Conversely, the administration of mahanimbine (1 mg/kg, p.o) significantly declined brain MDA levels in aged mice (7.56 ± 0.35 µM; *p* < 0.001) compared to the aged control. No considerable changes were noted with 2 mg/kg of mahanimbine (9.65 ± 0.51 µM).

The quantity of glutathione (GSH) in the brain homogenate suggested mahanimbine’s antioxidant potential. Statistical analysis of GSH (Figure 3B) showed a significant attenuation between the aged control group (0.0028 ± 0.0004 µmoles/mg; *p* < 0.001) when associated with the young control (0.0447 ± 0.0029 µmoles/mg). The present study also found that treatment with mahanimbine (1 and 2 mg/kg, p.o) reversed the GSH level, which was significantly higher (0.0372 ± 0.0038 µmoles/mg, *p* < 0.001 and 0.0282 ± 0.0041 µmoles/mg, *p* < 0.001; respectively) as matched to the aged control.

### 3.3. Mahanimbine Improved the Cholinergic Activity in the Aged Mouse Brain

**Figure 4:**
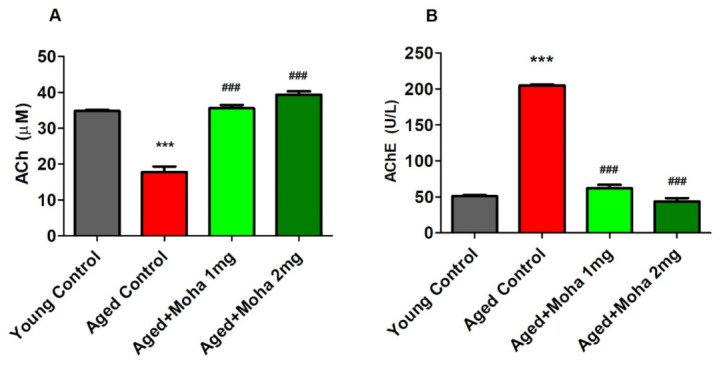
Effects of mahanimbine on cholinergic activity in aging mouse brain. (**A**) Comparison of the acetylcholine (ACh) level. (**B**) Comparison of the acetylcholinesterase (AChE) level. Mahanimbine treatment improved the cholinergic neuronal activities by elevating ACh levels and diminishing AChE activities. All data were expressed as mean ± SEM (*n* = 6). One-way ANOVA (*F*(3,20) = 85.1, *p* < 0.001 for ACh level and *F*(3,20) = 93.17, *p* < 0.001 for AChE level) followed by Tukey–Kramer multiple comparisons test. *** *p* < 0.001 in comparison with young control; ^###^ *p* < 0.001 in comparison with aged control.

Figure 4A shows the ACh levels in the mouse brain. ACh was comparably reduced (*p* < 0.001) in the aged control (17.79 ± 1.54 µM) as matched to the young control (34.82 ± 0.31 µM). Administration of mahanimbine (1 and 2 mg/kg), however, significantly increased (35.61 ± 0.85 µM, (*p* < 0.001); 39.42 ± 0.88 µM (*p* < 0.001); respectively) the level of ACh in the brain as associated with the aged control.

Figure 4B represents the effect of mahanimbine against ACHE activity in the brain. The activity of AChE in the aged control group (204.80 ±1.55 U/L) was considerably elevated (*p* < 0.001) as paralleled with the young control (51.06 ± 1.36 U/L). Nonetheless, the AChE level was suggestively inhibited with the treatment of 1 mg/kg (62.03 ± 4.64 U/L; *p* < 0.001) and 2 mg/kg (43.73 ± 4.76 U/L; *p* < 0.001) mahanimbine when associated with the aged control. Overall, administration of a high dose of mahanimbine (2 mg/kg, p.o) produced better results for cholinergic activity since it enhanced the ACh level and suppressed AChE activity.

### 3.4. Mahanimbine Inhibited Aβ1-40 and Aβ1-42 in the Aged Mouse Brain

**Figure 5:**
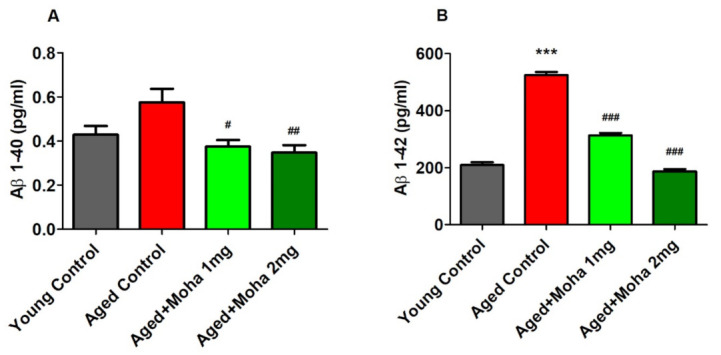
Effects of mahanimbine on the formation of β-amyloid (Aβ) levels in aged mouse brain. (**A**) Comparison of the Aβ_1-40_ level. (**B**) Comparison of the Aβ_1-42_ level. Mahanimbine administration significantly reduced Aβ levels in aged mouse brains. All data were expressed as mean ± SEM (*n* = 6). One-way ANOVA (*F*(3,20) = 5.696, *p* < 0.01 for Aβ_1-40_ level and *F*(3,20) = 50.50, *p* < 0.001 for Aβ_1-42_ level) followed by Tukey–Kramer multiple comparisons test. *** *p* < 0.001 in comparison with young control; ^#^ *p* < 0.05, ^##^ *p*< 0.01 and ^###^ *p* < 0.001 in comparison with aged control.

Figure 5A shows the effect of mahanimbine against the Aβ_1-40_ levels in brain homogenate of aged mice. There was no considerable difference in the Aβ_1-40_ level in aged control mice (0.58 ± 0.06 pg/mL) compared with young control mice (0.43 ± 0.04 pg/mL). Nevertheless, treatment with mahanimbine 1 mg/kg and 2 mg/kg significantly reduced (0.38 ± 0.03 pg/mL, *p* < 0.05; 0.35 ± 0.03 pg/mL, *p* < 0.01; respectively) the level of Aβ_1-40_ when linked with the aged control.

Figure 5B represents the potential of mahanimbine in suppressing the Aβ_1-42_ level in the brain of aged mice. The level of Aβ_1-42_ was considerably higher in the aged control (524.9 ± 11.11 pg/mL, *p* < 0.001) when related with the young control (209.2 ± 10.81 pg/mL). The treatment with mahanimbine 1 and 2 mg/kg, however, significantly reduced (313.2 ± 8.72 pg/mL, *p* < 0.001; 187.3 ± 7.97 pg/mL, *p* < 0.001; respectively) the level of Aβ_1-42_ when compared with the aged control group.

### 3.5. Mahanimbine Inhibited BACE-1 Activity and Expression in the Aged Mouse Brain

**Figure 6:**
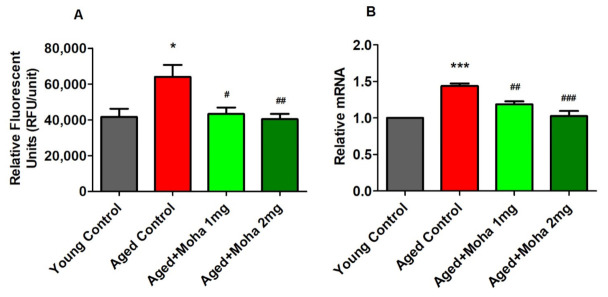
Effects of mahanimbine on BACE-1 in aging mouse brain. (**A**) Comparison of BACE-1 activity. (**B**) Comparison of the expression of the BACE-1 gene. Mahanimbine administration attenuated BACE-1 activity in aged mouse brain. All data were expressed as mean ± SEM *(n* = 6). One-way ANOVA (*F*(3,20) = 5.805, *p* < 0.01 for BACE-1 activity and *F*(3,20) = 21.46, *p* < 0.001 for expression of BACE-1 gene) followed by Tukey–Kramer multiple comparisons test. * *p* < 0.05 and *** *p* < 0.001 in comparison with young control; ^#^ *p* < 0.05, ^##^ *p* < 0.01, and ^###^ *p* < 0.001 in comparison with aged control.

Figure 6A highlights the effect of mahanimbine on BACE-1 activity. The activity of BACE-1 was significantly elevated in the aged control group (63,996 ± 6608 RFU/Unit; *p* < 0.05) compared with the young control (41,632 ± 4532 RFU/Unit). However, administration of 1 and 2 mg/kg of mahanimbine comparably inhibited (*p* < 0.05 and *p* < 0.01; respectively) the activity of BACE-1 when compared with the aged control group. The value of BACE-1 activity was 43,210 ± 3629 RFU/Unit in the 1 mg/kg mahanimbine group and 40,362 ± 2988 RFU/Unit in the 2 mg/kg mahanimbine-treated group.

To validate BACE-1 activity in the brain homogenate, expression of the BACE-1 gene was quantified using the RT-PCR method. Figure 6B displays the effect of mahanimbine on BACE-1 gene expression. Statistical analysis of BACE-1 expression in brain homogenate revealed that the aged control group displayed significantly higher BACE-1 expression (1.44 ± 0.03; *p* < 0.001) when related with the young control. Treatment with 1 and 2 mg/kg mahanimbine significantly reduced the expression of BACE-1 (1.19 ± 0.04 (*p* < 0.01), 1.03 ± 0.07 (*p* < 0.001); respectively) when matched with the aged control. From the findings, administration of a high dosage of 2 mg/kg mahanimbine showed greater prevention against BACE-1 activity and expression.

### 3.6. Effects of Mahanimbine on Total Cyclooxygenase (COX) Activity and COX-2 Expression in the Aged Mouse Brain

**Figure 7:**
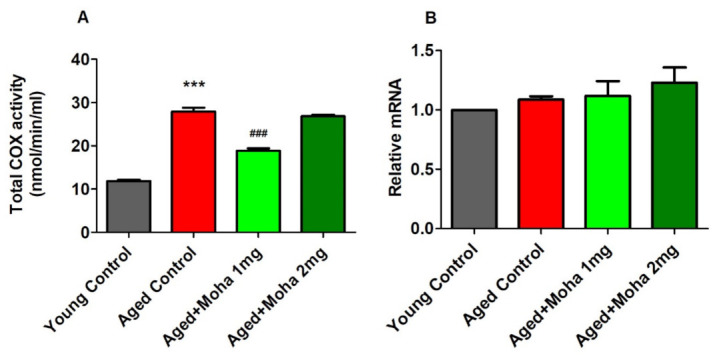
Effects of mahanimbine on COX activities in aged mouse brain. (**A**) Comparison of total COX activity. (**B**) Comparison of the expression of COX-2 gene. Mahanimbine administration ameliorated COX-2 activity in aged mouse brains. All data were expressed as mean ± SEM (*n* = 6). One-way ANOVA (*F*(3,20) = 78.20, *p* < 0.001 for total COX activity and *F*(3,20) = 1.096, *p* > 0.05 for expression of COX-2 gene) followed by Tukey–Kramer multiple comparisons test. *** *p* < 0.001 in comparison with young control; ^###^ *p* < 0.001 in comparison with aged control.

The ability of mahanimbine in suppressing neuroinflammation is indicated by the reduced activity of total COX activity in the brain homogenate. Figure 7A shows the total COX activity in the aged control group significantly increased (27.96 ± 0.89 nmol/min/mL; *p* < 0.001) compared with the young control (12.34 ± 0.07 nmol/min/mL). Treatment of mahanimbine at 1 mg/kg significantly reduced the total COX activity (18.87 ± 0.53 nmol/min/mL; *p* < 0.001) compared with the aged control, but there was no considerable difference with 2 mg/kg of mahanimbine (26.84 ± 0.37 nmol/min/mL) compared with the aged control.

COX-2 plays the main role in neuroinflammation; thus, the COX-2 gene was quantified using the RT-PCR method, and the data are presented in Figure 7B. Based on the statistical analysis of mRNA expression of the COX-2 gene, there were no notable differences between experimental groups (*F*(3,20) =1.096, *p* = 0.3737). The values for each group were: aged control group, 1.09 ± 0.03; young control group, 1.00 ± 0.00; 1 mg/kg mahanimbine group, 1.12 ± 0.13; and 2 mg/kg mahanimbine group, 1.23 ± 0.13.

## 4. Discussion

These study results have demonstrated that mahanimbine improves cognitive functions in aged mice. These conclusions specified that the administration of mahanimbine influenced learning and memory functions of aged mice via reduced oxidative stress, increased antioxidant level, improved cholinergic activity, attenuated deposition of Aβ, declined BACE-1 activity, and reduced total COX activity. It is widely accepted that aged rodents, like elderly humans, show aging-associated declines in cognitive functions with memory impairment [3,21,22].

Based on the MWM data in the present study, the thirty days’ treatment of mahanimbine improved memory parameters in aged mice since it decreased the EL and SD of mice as well as improved the time spent in the targeted quadrant compared with the aged control. Based on the average swimming speed of mice, there was no difference among groups. This indicated that the performance reduction in old mice was not caused by any perceptual deficiencies, such as vision or motor debits, and the performance gain produced by mahanimbine was not caused by swimming acceleration. From MWM findings, mahanimbine represents the first evidence of ameliorating cognitive deficits in aged mice.

An increase in oxidative stress is reported during aging. Oxidative stress is caused by an imbalance between ROS generation and elimination, which involves a significant role in age-related diseases [23]. Preventing oxidative stress and maintaining oxidative state is essential for optimal physiological function and preventing age-related diseases [24]. Antioxidants are considered a protective system in a rat’s brain while aging [25], and therefore, intake of antioxidant supplementation might protect the effects of ROS that inspire the progression of numerous chronic diseases [26]. Our present results verified the antioxidant effect of mahanimbine in aged mice. The decreased level of malondialdehyde (MDA) in the brain homogenate of aged mice treated with mahanimbine as matched to aged controls indicates the reversal of ROS activity in treated groups. MDA is one of the primary intermediates in free radical damage, which results in oxidative stress [27]. It also obstructs cerebral function by disrupting the balance of inhibitory and excitatory neurons in the brain [28]. Additionally, our results showed elevation in reduced glutathione (GSH) levels in mahanimbine-treated aged mice compared with aged control. These results specified that mahanimbine enhanced free radical scavenging functions in aged mice. This may be due to mahanimbine scavenging the activity of free radicals and restoring GSH levels. Based on a previous report, downregulation of GSH was apparent in the organs of aged rats matched to those of younger rats [29]. GSH is an endogenous antioxidant that protects against damage produced by oxygen-free radicals. Mahanimbine seems to have the ability to increase GSH levels to counter oxidative stress in the aged brain. Our present findings were consistent with previous reports that observed the antioxidative property of carbazole alkaloids from *M. koenigii* leaves [30]. These results strongly suggested that mahanimbine’s antioxidant potential was due to declining MDA levels in aged mice brains. Mahanimbine probably lowered MDA levels due to its antioxidant activity, which improved animal performance in the MWM task.

The enhanced memory in aged mice with mahanimbine treatment may also be due to improvement in central cholinergic transmission by increasing the acetylcholine (ACh) level and inhibiting the activity of acetylcholinesterase (AChE) in the aged mouse brain. Both ACh and AChE in the synapse were associated with the learning and memory functions [31]. Memory dysfunction is directly correlated with a decrease in the release of ACh at the neurons [32]. From a previous report, a 25% to 30% decrease in levels of ACh leads to severe memory loss in the AD animal model [33]. The present results highlighted a 50% decrease in the concentration of ACh in aged mice brains compared to young mice, which could have resulted in a more serious loss of cognitive function. However, treatment with mahanimbine recovered the ACh level in aged mice and led to an improvement in cognitive functions. One of the important strategies to elevate the cholinergic function is by inhibiting AChE. This involves the breakdown of ACh into choline in the neural synapse, which causes cholinergic deficit and contributes to cognitive impairment [34]. Based on the AChE data, treatment with mahanimbine significantly decreased AChE activity in the aged mouse brain and concurrently increased the concentration of ACh in the brain. This finding is parallel with the study by Kumar et al. (2010) [35] in that inhibition of AChE helps to enhance ACh activity, which is one of the main approaches in the management of AD.

On the other hand, it is well known that β-amyloid (Aβ) is continuously synthesized from its precursor and catabolized under normal settings, whereas the aging process leads to the pathological deposition of Aβ, which results from a defective metabolism [36]. Accumulation of Aβ induced abnormalities of neuronal function, thus resulting in cognitive dysfunction [37]. Consumption of dietary supplements can help reduce aging-related Aβ accumulation, which is a key method for preventing cognitive impairment. Therefore, the amount of Aβ_1-40_ and Aβ_1-42_ has been measured in brain homogenate of aged mice supplemented with mahanimbine. The Aβ_1-40_ denotes the most abundant isoform in brain tissue, whereas Aβ_1-42_ shows a considerable increase in the brain of AD patients [38]. The level of Aβ_1-40_ and Aβ_1-42_ were significantly decreased in the mahanimbine-treated group of aged mice compared to the aged control. Additionally, the present study also evaluated the activity of BACE-1, which is an enzyme that contributes to the formation of Aβ [39]. Aβ was produced from APP by proteolytic cleavage by β-secretase (BACE-1) and followed by γ-secretase [40]. The generation of Aβ was initiated by BACE-1; it metabolizes APP to APPβ, Aβ N terminus, and a C-terminal fragment, C99. Then, γ-secretase produces Aβ [41]. The current study showed that oral administration of mahanimbine in aged mice declined the activity of BACE-1 compared with an aged control. The gene expression of BACE-1 was also lower with the administration of mahanimbine, further confirming the results obtained. Thus, decreased activity of BACE-1 and expression of the BACE-1 gene consequently reduced the formation of Aβ and significantly attenuated spatial learning and memory deterioration.

The present study also studied the effect of mahanimbine on inflammatory markers, which were total COX activity and COX-2 expression. From our finding, aged mice showed a high level of total COX activity, but treatment with mahanimbine had the capability to attenuate total COX activity at only the 1 mg/kg level. However, expression of the COX-2 gene in aged mice brains did not exhibit any notable differences as matched to young controls. There are several epidemiological findings that link inflammation and aging to predict a variety of aging phenotypes, including changes in neuronal health, metabolic homeostasis, body composition, and immune senescence [5].

The present study has some limitations; recently, a few reports have highlighted that anti-inflammatory agents do not support AD management. Moreover, this initial evaluation resulted in using a mouse model, where some discrepancy between mice and humans might be due to the different functions of microglial cells, particularly microglial cells triggering the inflammatory process and aggravating AD, but they neither express iNOS nor produce NO, but protect neurons via growth factors (GF) and neurotrophic factors (NF) in humans [42]. However, the current results support the further evaluation of mahanimbine on more specific targets, including aging-related memory functions.

## 5. Conclusions

Overall, the present study indicates that mahanimbine could improve spatial learning and memory since it showed shorter EL and SD and enhanced time spent in the targeted quadrant during the MWM test in aged mice. In biochemical analysis, mahanimbine decreased the levels of MDA, AChE, Aβ_1-40,_ Aβ_1-42_, BACE-1, and total COX but raised the level of GSH and ACh in aged mice’s brains compared to aged controls. In conclusion, mahanimbine could protect learning and memory impairment in aged mice through attenuation of oxidative stress (MDA), deposition of Aβ_1-42_, AChE level, and BACE-1 activity while increasing antioxidant (GSH) and ACh levels. Therefore, mahanimbine could be a potential substitute for treating aging-related conditions. However, the mechanistic aspect of the neuroprotective effect of mahanimbine for improving cognitive function needs to be further evaluated.

## Data Availability

The data presented in this study are available from the corresponding author upon reasonable request.
